# Autism and education—international policy in small EU states: policy mapping in Malta, Cyprus, Luxembourg and Slovenia

**DOI:** 10.1093/eurpub/ckaa146

**Published:** 2020-09-03

**Authors:** Robin van Kessel, Rok Hrzic, Katarzyna Czabanowska, Aurélie Baranger, Natasha Azzopardi-Muscat, Nefi Charambalous-Darden, Carol Brayne, Simon Baron-Cohen, Andres Roman-Urrestarazu

**Affiliations:** c1 Department of International Health, Faculty of Health, Medicine and Life Sciences, Maastricht University, Maastricht, The Netherlands; c2 Department of Health Policy Management, Institute of Public Health, Faculty of Health Care, Jagiellonian University, Krakow, Poland; c3 National Institute of Public Health, Warsaw, Poland; c4 Autism-Europe, Brussels, Belgium; c5 Department of Health Services Management, Faculty of Health Sciences, University of Malta, Msida, Malta; c6 Islands and Small States Institute, WHO Collaborating Centre on Health Systems and Policies in Small States, University of Malta, Msida, Malta; c7 Centre for Education and Research, University of Northampton, Northampton, UK; c8 Institute of Public Health, University of Cambridge, Cambridge, UK; c9 Department of Psychiatry, Autism Research Centre, University of Cambridge, Cambridge, UK

## Abstract

**Background:**

Special education provides an array of support that can advantageously meet special education needs (SEN) of children with autism. This report maps autism and SEN policies, and tension of international legislation in Malta, Cyprus, Luxembourg and Slovenia.

**Methods:**

A policy path analysis was performed using a scoping review as fundamental methodological framework.

**Results:**

Education for children with SEN developed from limited education towards segregation, and further to integration, and inclusion in mainstream education. International policy has greatly influenced the education systems under study. The rights to education and to have SEN addressed have been adopted in all countries. Inclusion is seen to be gradually incorporated by Malta, Cyprus and Luxembourg—closely following values of international documents through concise SEN policies. Slovenia’s education system remains segregated, indicating potential tension.

**Conclusions:**

It appears that mainstream schools offer SEN services until no longer feasible for the child in the majority of investigated countries. Inclusion has become a guiding principle for most education systems under study. Finally, small states either commit to the implementation of inclusion or delay it and attempt to improve the education system for children with SEN in different ways.

## Introduction

Several studies have investigated the tension between big and small European Union (EU) Member States in their approach towards European involvement in aspects of policy traditionally left to Member States including health and education.^1–4^ Small states, in this context, are defined as states that not only have a small population size, but also are not in a position to influence the international policy environment on their own and are, by extension, largely dependent on the decisions of larger states and overarching political structures.

With the respective adoption of the Universal Declaration of Human Rights (UDHR) in 1948 and the Convention of the Rights of Persons with Disabilities (CRPD) in 2006,^5^^,^^6^ the right to education for children with special education needs (SEN) internationally has been well established. Its values of the UDHR have significantly influenced the course of disability and education policy in the previously investigated countries. The CRPD covers people with disabilities and it highlights that children with disabilities need to be able to fully enjoy all human rights and fundamental freedoms equally to other children. As a result, it cements any future development of children and people with disabilities and their right to education. However, implementing this right with respect to access to education for children with autism in the EU has been challenging.^7^ With a male-to-female ratio between 3:1 and 4:1, autism affects roughly 1% of the population.^8^^,^^9^ Autistic people may be more susceptible to severe health and other functional difficulties that may result in financial problems for families and caregivers and the condition can still carry considerable stigma.^10–13^ To address these circumstances and increase the inclusion and quality of life of autistic people and the autism community across Europe, the application of fundamental rights of education is paramount.^14^^,^^15^

It is pivotal for EU Member States to provide adequate SEN services starting from early childhood and continuing throughout school years, while simultaneously supporting autistic people in life-long education.^8^^,^^16^ The evidence base of SEN support has been reported previously:^7^ support in cognition and learning; social, emotional and mental health; and communication and interaction could lead to significant benefits for the development of children with SEN, while also acknowledging that the SEN for children with autism may differ significantly per person. It also emphasized the importance of children having their SEN met in education settings. A recent example of a novel approach to SEN that has been adopted by schools is inclusive education,^15^ in which the required SEN services are provided in a mainstream education setting, enabling children with SEN to engage in education along with typical peers. Providing these services adequately can result in major benefits for a child’s cognitive and social development.

This article focuses on mapping SEN and autism policy in Malta, Cyprus, Luxembourg and Slovenia. These countries make up four out of the five smallest EU Member States and are therefore deemed suitable to fill the definition of small state.^17^ Since small states often experience tension when dealing with the implementation of international policies, the second aim of this article is to investigate the extent of this tension in the area of autism and special education policy. It should be noted that, even though Estonia fits the definition of a small state, it was not included as it joined the UN significantly later (1991) compared to the other countries under study [Malta 1964, Cyprus 1960, Luxembourg 1945 and Slovenia 1945 (as part of former Yugoslavia)] and had been under communist influence prior to its independence.^18^ Determining the influence of the communist era on the national policy environment on top of investigating possible tensions would be beyond the scope of this study.

## Methods

The policy mapping framework in this article is based on previously validated work.^19–21^ Data were gathered through a scoping review and analyzed through a policy path dependence analysis.^22–24^ Due to the absence of a comprehensive EU data source on autism and SEN policy, a modular approach was adopted to analyze the education policy environments under study. The findings were reported using the PRISMA framework.^25^

### Eligibility criteria

Consistent with previous work,^19–21^ inclusion criteria consisted of (i) a scope relating to the right to education, national education system, disability laws, inclusion or SEN; (ii) aimed at children under 18 years; (iii) drafted by a governmental institution; and (iv) published after 1948. Constitutions were always included and no language limitations were set. Non-governmental policies and actions were excluded.

### Data collection and search strategy

Like previous work,^19–21^ the data collection consisted of five steps in which governmental websites formed the primary source for data collection: (i) review and extract policies relevant to the education of children with SEN; (ii) develop a multi-layered search strategy for scientific databases (Google Scholar/PubMed); (iii) merge policy and academic publications conform the eligibility criteria; (iv) acquire further information through searching reference lists; and (v) merge all documents into one data repository for the purpose of this scoping review and path dependence analysis.

Table 1 shows the policy repositories that were used per country. The search strategy involved searching full-texts for the respective keywords, instead of just titles and abstracts in order to minimize the risk of overlooking policy as a result of inaccurate or incomplete translations. The used keywords consisted of: autism, disability, SEN, education, special needs, special education and inclusive education. These were translated into French (Luxembourg), Greek (Cyprus) and Slovenian, respectively, and used individually, as combining the keywords in the policy repositories yielded little relevant results. Subsequently, the exact build-up of the search query used for scientific databases is shown in table 2. The data collection took place between 2 February and 9 April 2019. Sources from a later date were added as a result of consulting country experts.

**Table 1:** The policy databases used per country

Country	Link
All	http://eur-lex.europa.eu/n-lex/
Malta	http://justiceservices.gov.mt; https://education.gov.mt/en/education/student-services/Pages/default.aspx; http://www.legislation.mt/
Cyprus	http://www.cylaw.org
Luxembourg	http://legilux.public.lu/
Slovenia	http://www.pisrs.si/Pis.web/#

**Table 2:** The build-up of the final search query for academic databases

	Search query
Term 1	((((((((((autism & law) OR autism & policy) OR autism & SEN) OR autism & education) OR autism & disability) OR SEN & policy) OR SEN & law) OR disability & law) OR disability & policy))
Term 2	(Malta OR Cyprus OR Luxembourg OR Slovenia)
Final query	((((((((((autism & law) OR autism & policy) OR autism & SEN) OR autism & education) OR autism & disability) OR SEN & policy) OR SEN & law) OR disability & law) OR disability & policy)) AND ((Malta OR Cyprus OR Luxembourg OR Slovenia))

### Data analysis

Gathered data were compared to data on UN and EU policy in previous work.^19–21^ As such, the extent to which the values of international policies are integrated in the national policies could be established. To gauge possible tension in the small Member States, we tracked the implementation of various values that were set out in international policy: (i) the universal right to education laid down in the UDHR; (ii) the right for children to receive appropriate treatment consistent with their condition by the Declaration on the Rights of the Child; (iii) the right for children with developmental, intellectual and learning conditions to receive appropriate education to maximize their potential by the Declaration on the Rights of Disabled Persons; and (iv) the development of an inclusive education environment as set out by the Salamanca Statement and the CRPD. Lack of implementation of any of these points is considered tension.

## Results

**Figure 1:**
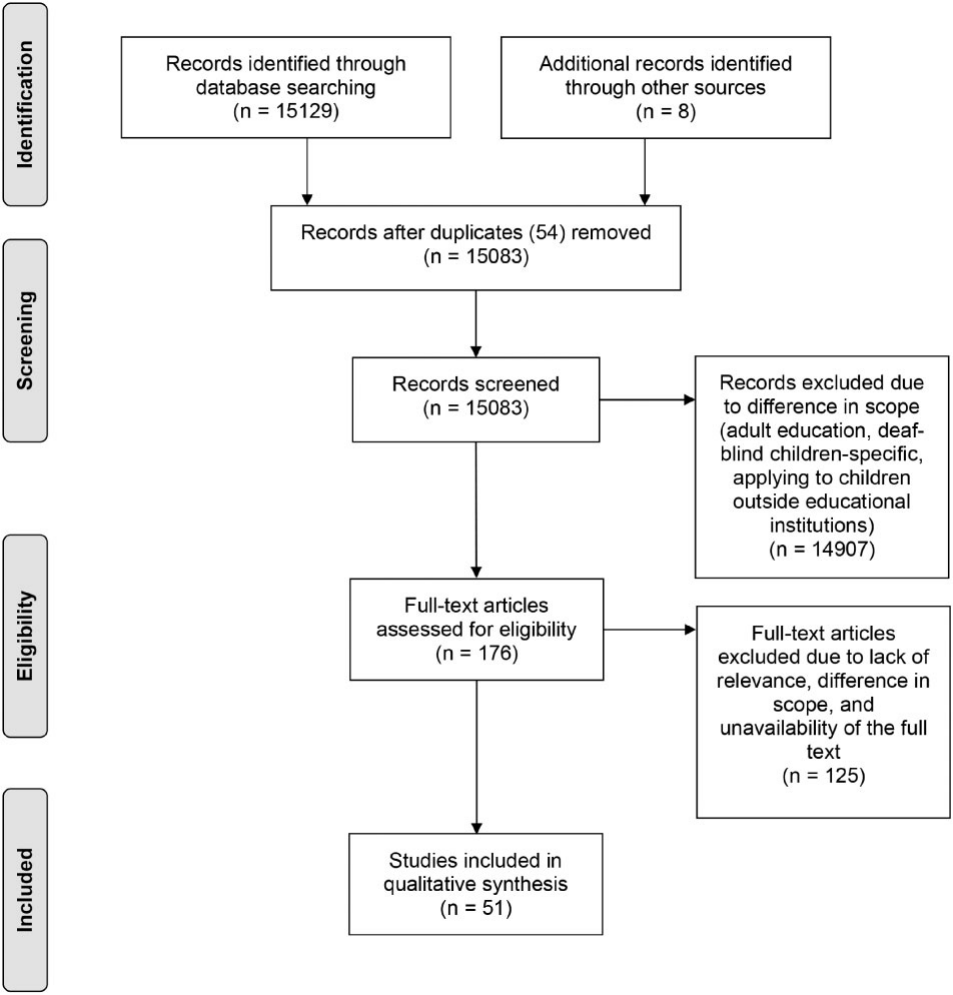
A visualization of the process that was used to select relevant articles from the search strategy

We identified 15 129 sources (94 for Malta, 5583 for Cyprus, 2622 for Luxembourg and 6830 for Slovenia) through database searching and 7 through other sources. CyLaw returned individual chapters, rather than complete documents. As such, the entire policy document was included if two separate chapters of the same document were identified by the search query. A total of 51 documents (8 for Malta, 17 for Cyprus, 9 for Luxembourg and 17 for Slovenia) were ultimately included in this review. A PRISMA flowchart illustrates the entire process in figure 1. A synopsis of the policy contents is included in Supplementary file S1. Supplementary file S2 shows the policy and academic documents that were used per country.

### Malta

The development of Maltese SEN policy is divided into two categories: the development of the education system and the empowerment of people with disabilities. Firstly, the development of the education system is covered by the Education Act (1988), creating an environment where all children were guaranteed primary education. It provided a definition of SEN—a child that has particular difficulties of physical, intellectual, sensory or psychological essence—and established Resource Centres, aimed to educate children with SEN who were unable to follow mainstream education, effectively ensuring a place for every child in the education system. Secondly, the empowerment of people with disabilities is achieved through the Equal Opportunities (Persons with Disabilities)-, Mental Health-, and Persons within the Autism Spectrum (Empowerment) Act of 2000. While the former two heavily focus on establishing rights for people with disabilities, the latter expanded on this by formulating clear guidelines on how to improve the environment for people with autism in terms of (i) improving the education of pupils with autism; (ii) informing people close to the person with autism; and (iii) raising awareness. Malta adopted a new Education Act in 2019, in which the right to education was re-emphasized. It also expanded the environment of inclusion by requiring teachers to keep developing themselves professionally and create collaborations between teachers, children and parental committees as to ensure high-quality education.

In Malta, autism falls under inclusive education policy and the Autism Spectrum Support Service is specifically geared towards children with autism.^26^ It recognizes that each student is an individual, and seeks to maximize the student’s potential.^27^ It aims to empower educators and parents to meet the individual education needs of the child with autism, facilitating learning, supporting behavioural and social development and maximizing on students’ abilities in mainstream and resource environment. Additionally, a national early screening programme for autism has been established.^28^ It is a simple means to help identify possible developmental delays, learning difficulties and/or education needs related to autism so children can be supported before starting kindergarten. Furthermore, it involves a process that starts at the age of 18 months through M-CHAT—a short questionnaire, which is carried out with the parents or guardians and helps provide an indication of any risks relating to autism. The development of an inclusive education system in Malta also does not signal tension and is in line with internationally available guidance.

### Cyprus

The first special school was established in 1929 and by 1979 all special schools were established.^29^ A draft legislation was initiated in 1979 to monitor issues regarding special schools.^30^ This draft resulted in a new law that legalized the operations of special schools: The Special Education Act.

The Education and Training of Children with Special Needs Act of 1999 was essential for the Cypriot education system,^31^ as children with SEN were no longer only educated in special schools, but also placed in more specialized institutions. The Education and Training of Children with Special Needs Act passed in 1999 came in effect in 2001, which resulted in a split system of education. Only the children with mild-to-moderate SEN were placed into mainstream school settings, while children with more severe SEN were separated and placed in special schools or institutions. These remained unprepared to accept children with more severe SEN due to lacking infrastructure to ensure quality education.^29^^,^^32^^,^^33^

Jones and Symeonidou^31^ elaborate on the Code of Practice for Referring Children with Special Needs to the Special Education and Training District Committees that aims to motivate schools and teachers to work with children that are suspected to have SEN before referring them for assessment, and work on early detection mechanisms for SEN. Inclusion was introduced in the 2014 amendments to the Education and Training of Children with Special Needs Act. No distinction between various disabilities is made though. Therefore, no specialized provisions for autism specifically are established. Circulars in 2016, 2018 and 2019 targeted to improve education and train teachers in working with children with autism—further solidifying their right to receive appropriate education. Ultimately, education in Cyprus has evolved from a segregated system towards a system of inclusion in line with international guidance, indicating no tension.

### Luxembourg

Policies until 1994 developed the education system in Luxembourg so that every child can follow a form of education. Compulsory education required all children to receive nine consecutive years of education—in special classes if necessary. The introduction of differentiated education allowed children that could not function in special classes to receive education according to their capabilities. In this education setting, teachers were assisted by an array of supporting staff.

Policies after 1994 focussed on the development of more inclusive practices. Children with SEN had the option to attend either mainstream education, in which case individualized care plans were implemented to help adjust, special education, or differentiated education. Furthermore, specialized centres were implemented to aid with the uptake of children with SEN in the education system by aiding the personal development of the child and the schools.

Ultimately, the education system in Luxembourg is regulated in such a way that there is room for every child in the system. They initially implemented a segregated education environment through differentiated education, although this has evolved towards a more integrated/inclusive approach that is in line with international guidance, while retaining the option of segregated schools in case a child cannot participate in mainstream schools.

### Slovenia

The initial focus of the Slovenian education system, which encompasses the policies up to 2005, was the development of a non-discriminatory environment where all children have a place. Children with SEN followed a modified version of the mainstream curriculum that was adapted to their needs. These adaptations could be in the form of additional professional assistance, as well as adaptation of class sizes—ranging between 4 and 12 pupils per class depending on the severity of their conditions. Modified education programmes were also made available for children with SEN to use.

Policies from 2010 onwards intend to develop SEN education to be on par with education for typical children—to the point a tailored primary school programme of equivalent education standards is created for children with autism. There is a small notion of inclusion of children with SEN in education systems and that children with SEN should be guided to the most suitable education programme. Overall, policies work towards the improvement of the existing segregated system, though never progressing towards and integrative or inclusive environment.

In short, the education system in Slovenia has seen a development of their education system that is consistent with the values set out by most respective international documents—the only point of tension being the move towards inclusion. A unique element in the Slovenian system is the elaborate regulation on class sizes depending on the condition of the children. Numerous policies were implemented specifically in an attempt to equalize and/or unify the values of educational achievements in mainstream and special education.

## Discussion

This study aimed to map autism and SEN policies in Malta, Cyprus, Luxembourg and Slovenia and to investigate how international SEN and human right policy was addressed at the national policy level in these small states. All relevant SEN policies that affect the education of children with autism and their universal right to education were mapped.

The UDHR formed a critical juncture in international, EU and national policy.^19–21^ Integration and inclusion became guiding principles in most countries—except Slovenia. Legislation was found to address discrimination of people on basis of disability. International documents greatly influenced the development of national education systems—most notably the right to education for all children, the right to have SEN adequately addressed and the ideology of inclusive education have been adopted by most countries under study.

An inference was made previously that *health* reforms in small states are predominantly lead by shifting ideologies over supranational influence.^1^^,^^2^ However, *education* policy development closely follow the values set out in guiding international documents and their approaches also resemble the actions of larger EU Member States—particularly regarding inclusive education and allocating responsibilities to address SEN to schools and teachers.^21^ Finally, almost no policy across the countries under study establishes or recommends specific tools or tool requirements, except for Malta.^28^

Some cross-country differences were found. Malta was the only country that had adopted policy, aiming to include children with autism specifically in the education system. While the others adopted their own policy in enabling children with SEN to access education, the scope of the Maltese legislation expanded beyond that—intending to fully integrate autistic people in society on par with typical people, focussing health, well-being, participation and striving for autonomy for their autism community. Moreover, Malta implemented specific services aiding educators and parents in addressing the needs of a child with autism and an autism-specific screening programme that starts at the age of 18 months. Since the earliest symptoms of autism generally appear between the age of 6 and 12 months,^8^ this screening programme can benefit early identification and early intervention for children with autism. Slovenia prescribed class sizes of primary, secondary and special education programmes based on SEN categories that would be present in-class, along with possibilities of home schooling. Organizations for people with disabilities were introduced, promoting their human rights, non-discrimination and equal opportunities. Interestingly, Slovenia adopted a higher number of education policies that pertain to SEN. A possible explanation for this is that Slovenian policies tend to introduce small/incremental changes, whereas the other countries’ policies introduce larger-scale changes. While no approach is inherently superior to the other, the Slovenian approach may run the risk of becoming too specific, which could potentially lead to the fragmentation of education for children with different SEN. Finally, no policy initiatives were found in Cyprus to increase awareness of SEN, autism or mental health in general. Regardless, it is unknown to what extent mental health awareness is already being raised in the community by non-governmental organizations (NGOs) for example.

Compared to larger countries, the findings of this study indicate that, during the development of the respective education systems, small states tend to establish separate institutions that tend to the various needs of children with SEN, including health and education—going beyond the scope of special schools that were commonly established in larger countries.^19–21^ This tendency was also found in the German-speaking community in Belgium, which holds the characteristics of a small state.^20^ Additionally, small states either commit to the implementation of inclusive education as soon as possible (Malta, Cyprus and Luxembourg) or delay it and attempt to improve the education environment for children with SEN in other ways (Slovenia), whereas larger countries tend to adopt a gradual implementation in which the system in-place slowly shifts towards inclusion.^19^^,^^20^

This scoping review has some limitations that should be accounted for. Results of this study cannot be generalized beyond the investigated countries; outcomes of this study remain strictly theoretical as the practical situation was not investigated (for instance the findings of Cyprus insinuate that children have not been properly assessed on whether they have SEN when they come to school, which would be crucial to assess in-practice); the possibility of errors in translation or misinterpretation cannot be dismissed—though Maltese, Cypriot, Luxembourgian and Slovenian experts were involved to assist interpreting the legislation for the respective countries; only governmental documents were included in this research, disregarding actions belonging to NGOs; the terms ‘autistic’ and ‘disabled’ were not included in the search terms—though this is ameliorated by involving country experts and the search strategy using full-texts rather than titles only; and the scope of the search strategy of this study was strictly limited to children—meaning individuals under the age of 18. Adult education was not covered, even though similar challenges with SEN may be present there.

Ultimately, this study provided insight in the SEN policy environment of Malta, Cyprus, Luxembourg and Slovenia. Path dependency analysis indicates the integration of most values of the UDHR, CRPD and other international documents in their education systems through national legislation. Mainstream schools offer SEN services and support until children cannot participate in mainstream education. At that point, specialized institutions are in charge of the education of the children. Inclusive education has become a guiding factor in the education systems of most countries under study. Finally, all countries under study account for children with autism in their respective education policy—with Slovenia retaining segregation, while Malta, Cyprus and Luxembourg work towards inclusion.

## Supplementary data

Supplementary data are available at *EURPUB* online.

## Supplementary Material

ckaa146_supplementary_data

## References

[1] Azzopardi-Muscat N, Funk T, Buttigieg SC, et al Policy challenges and reforms in small EU member state health systems: a narrative literature review. Eur J Public Health 2016;26:916–22. 27335326 10.1093/eurpub/ckw091

[2] Azzopardi-Muscat N, Sorensen K, Aluttis C, et al Europeanisation of health systems: a qualitative study of domestic actors in a small state. BMC Public Health 2016;16:334. 27079508 10.1186/s12889-016-2909-0 PMC4832556

[3] Thorhallsson B, Wivel A Small states in the European Union: what do we know and what would we like to know? Cambridge Rev Int Aff 2006;19:651–68.

[4] Haugevik K, Rieker P Autonomy or integration? Small-state responses to a changing European security landscape. Glob Aff 2017;3:211–21.

[5] United Nations. Universal Declaration of Human Rights, 1948 Available at: http://www.un.org/en/udhrbook/pdf/udhr_booklet_en_web.pdf (23 August 2020, date last accessed)

[6] United Nations. Convention on the Rights of Persons with Disabilities, 2006 Available at: http://www.ohchr.org/EN/HRBodies/CRPD/Pages/ConventionRightsPersonsWithDisabilities.aspx#3 (23 August 2020, date last accessed)

[7] Carroll J, Bradley L, Crawford H, et al SEN support: a rapid evidence assessment, 2017.

[8] Lai M-C, Lombardo MV, Baron-Cohen S Autism. Lancet 2014;383:896–910. 24074734 10.1016/S0140-6736(13)61539-1

[9] Loomes R, Hull L, Mandy WPL What is the male-to-female ratio in autism spectrum disorder? A systematic review and meta-analysis. J Am Acad Child Adolesc Psychiatry 2017;56:466–74. 28545751 10.1016/j.jaac.2017.03.013

[10] Knapp M, Romeo R, Beecham J Economic cost of autism in the UK. Autism 2009;13:317–36. 19369391 10.1177/1362361309104246

[11] Howlin P, Goode S, Hutton J, Rutter M Adult outcome for children with autism. J Child Psychol Psychiatry 2004;45:212–29. 14982237 10.1111/j.1469-7610.2004.00215.x

[12] van Heijst BFC, Geurts HM Quality of life in autism across the lifespan: a meta-analysis. Autism 2015;19:158–67. 24443331 10.1177/1362361313517053

[13] World Health Organisation. Autism Spectrum Disorders, 2017 Available at: http://www.who.int/mediacentre/factsheets/autism-spectrum-disorders/en/ (25 April 2018, date last accessed).

[14] European Commission. European Disability Strategy 2010-2020: A Renewed Commitment to a Barrier-Free Europe, 2010 http://eur-lex.europa.eu/LexUriServ/LexUriServ.do?uri=COM:2010:0636:FIN:en:PDF (20 July 2020, date last accessed)

[15] Hehir T, Grindal T, Freeman B, et al A Summary of the Research Evidence on Inclusive Education. São Paulo: Instituto Alana, 2016 Available at: https://www.abtassociates.com/sites/default/files/2019 03/A_Summary_of_the_evidence_on_inclusive_education.pdf (3 July 2020, date last accessed)

[16] Baron‐Cohen S Editorial Perspective: neurodiversity–a revolutionary concept for autism and psychiatry. J Child Psychol Psychiatry 2017;58:744–7. 28524462 10.1111/jcpp.12703

[17] Eurostat. Population Change - Demographic Balance and Crude Rates at National Level, 2018 Available at: http://appsso.eurostat.ec.europa.eu/nui/show.do? dataset=demo_gind&lang=en (30 April 2018, date last accessed).

[18] United Nations. Growth in United Nations Membership, 1945-Present, 2020 Available at: https://www.un.org/en/sections/member-states/growth-united-nations-membership-1945-present/index.html (5 July 2020, date last accessed).

[19] Roleska M, Roman-Urrestarazu A, Griffiths S, et al Autism and the right to education in the EU: policy mapping and scoping review of the United Kingdom, France, Poland and Spain. PLoS One 2018;13:e0202336. 30161146 10.1371/journal.pone.0202336 PMC6116926

[20] van Kessel R, Roman-Urrestarazu A, Ruigrok A, et al Autism and family involvement in the right to education in the EU: policy mapping in the Netherlands, Belgium and Germany. Mol Autism 2019;10:43. 31827745 10.1186/s13229-019-0297-x PMC6902602

[21] van Kessel R, Walsh S, Ruigrok AV, et al Autism and the right to education in the EU: policy mapping and scoping review of Nordic countries Denmark, Finland, and Sweden. Mol Autism 2019;10:44. 31867091 10.1186/s13229-019-0290-4 PMC6907142

[22] Arksey H, O'Malley L Scoping studies: towards a methodological framework. Int J Soc Res Methodol 2005;8:19–32.

[23] Levac D, Colquhoun H, O’Brien KK Scoping studies: advancing the methodology. Implement Sci 2010;5:69. 20854677 10.1186/1748-5908-5-69 PMC2954944

[24] Mahoney J Path dependence in historical sociology. Theory Soc 2000;29:507–48.

[25] Moher D, Liberati A, Tetzlaff J, Altman DG; PRISMA Group. Preferred reporting items for systematic reviews and meta-analyses: the PRISMA statement. BMJ 2009;339:b2535. 19622551 10.1136/bmj.b2535 PMC2714657

[26] Spiteri L, Borg G, Callus AM, et al Inclusive and Special Education Review. Floriana, 2005 Available at: https://education.gov.mt/en/resources/Documents/Policy Documents/inclusive and special education review.pdf (22 August 2019, date last accessed).

[27] Government of Malta. Autism Spectrum Support Team, 2016 Available at: https://education.gov.mt/en/education/student-services/Pages/Inclusive_Education/Autism-Spectrum-Support-Team.aspx (22 August 2019, date last accessed).

[28] Government of Malta. Lenti fuq l-Iżvilupp ta` Wliedna, 2016 Available at: https://education.gov.mt/en/Lenti/Pages/Lenti.aspx (22 August 2019, date last accessed).

[29] Phtiaka H Educating the other: a journey in Cyprus time and space In: Len Barton L, Armstrong F (eds). *Policy, Experience and Change: Cross-Cultural Reflections on Inclusive Education* Dordrecht: Springer, 2008: 147–61.

[30] Symeonidou S Parental involvement in education politics: the case of disabled children. Mediterr J Educ Stud 2007;12:45–67.

[31] Jones C, Symeonidou S The Hare and the Tortoise: a comparative review of the drive towards inclusive education policies in England and Cyprus. Int J Incl Educ 2017;21:775–89.

[32] Hadjikakou K, Petridou L, Stylianou C The academic and social inclusion of oral deaf and hard‐of‐hearing children in Cyprus secondary general education: investigating the perspectives of the stakeholders. Eur J Spec Needs Educ 2008;23:17–29.

[33] Liasidou A Inclusive education policies and the feasibility of educational change: the case of Cyprus. Int Stud Sociol Educ 2007;17:329–47.

