# Supplementary material for: Autism and education—international policy in small EU states: policy mapping in Malta, Cyprus, Luxembourg and Slovenia
Source: Eur J Public Health. 2020 Sep 3;30(6):1078–83. doi: 10.1093/eurpub/ckaa146 (PMC7733051; doi:10.1093/eurpub/ckaa146)
Supplement: ckaa146_supplementary_data [file ckaa146_supplementary_data.zip › ejph-2020-01-om-0079-File004.docx]

**Supplementary File 2. A list of all policy and academic sources used per country.**

**Malta**

1. Maltese Ministry for Justice, Culture, and Local Government. Education Act (Cap. 327). 1988. Available from: http://www.justiceservices.gov.mt/DownloadDocument.aspx?app=lom&itemid=8801&l=1
2. Maltese Ministry for Justice, Culture, and Local Government. Equal Opportunities (Persons with Disability) Act (Cap. 413). 2000. Available from: http://www.justiceservices.gov.mt/DownloadDocument.aspx?app=lom&itemid=8879&l=1
3. Maltese Ministry for Justice, Culture, and Local Government. Mental Health Act (Cap. 525). 2013. Available from: http://www.justiceservices.gov.mt/DownloadDocument.aspx?app=lom&itemid=11962&l=1
4. Maltese Ministry for Justice, Culture, and Local Government. Persons within the Autism Spectrum (Empowerment) Act (Cap. 557). 2016. Available from: http://www.justiceservices.gov.mt/DownloadDocument.aspx?app=lom&itemid=12505&l=1
5. Spiteri L, Borg G, Callus AM, Cauchi J, Sciberras M. Inclusive and Special Education Review. Floriana; 2005 [cited 2019 Aug 22]. Available from: https://education.gov.mt/en/resources/Documents/Policy Documents/inclusive and special education review.pdf
6. Government of Malta. Autism Spectrum Support Team. 2016 [cited 2019 Aug 22]. Available from: https://education.gov.mt/en/education/student-services/Pages/Inclusive_Education/Autism-Spectrum-Support-Team.aspx
7. Government of Malta. Lenti fuq l-Iżvilupp ta` Wliedna. 2016 [cited 2019 Aug 22]. Available from: https://education.gov.mt/en/Lenti/Pages/Lenti.aspx
8. Maltese Ministry of Education. Education Act. 2019. Available from: https://legislation.mt/eli/act/2019/29/eng/pdf Accessed 5 July 2020.

**Cyprus**

1. Republic of Cyprus. Constitution of Cyprus. 1960. Available from: http://www.cylaw.org/nomoi/arith/1960_1_011_TKS.pdf
2. Phtiaka H. Educating the Other: A Journey in Cyprus Time and Space. In: Policy, Experience and Change: Cross-Cultural Reflections on Inclusive Education. Dordrecht: Springer Netherlands; 2008 [cited 2019 Aug 29]. p. 147–61. Available from: http://link.springer.com/10.1007/978-1-4020-5119-7_11
3. Symeonidou S. Parental involvement in education politics: the case of disabled children. Mediterr J Educ Stud. 2007 [cited 2019 Aug 20];12(2):45–67. Available from: https://gnosis.library.ucy.ac.cy/handle/7/38584
4. Cypriot Ministry of Education and Culture. Special Education Act. 1979. Available from: http://www.cylaw.org/nomoi/arith/1979_1_047.pdf
5. N. 113(I)/99. Integration of Children with Special Needs Act. Ofﬁcial Gaz Repub Cyprus. 1999;
6. Jones C, Symeonidou S. The Hare and the Tortoise: a comparative review of the drive towards inclusive education policies in England and Cyprus. Int J Incl Educ. 2017 Jul 3 [cited 2019 Aug 19];21(7):775–89. Available from: https://www.tandfonline.com/doi/full/10.1080/13603116.2017.1283715
7. Cypriot Ministry of Education and Culture. Rules on the Education and Training of Children with Disabilities (186/2001). 2001 [cited 2019 Aug 31]. Available from: http://www.cylaw.org/KDP/data/2001_1_186.pdf
8. Cypriot Ministry of Education and Culture. The Early Detection Mechanism for Children with Special Needs Regulations (185/2001). 2001 [cited 2019 Aug 31]. Available from: http://www.moec.gov.cy/eidiki_ekpaidefsi/nomothesia/peri_mihanismou_kanonismoi_2001_185_2001.pdf
9. Republic of Cyprus. Education and Training of Children with Special Needs Act (N. 113(I)/1999). Nicosia: Republic of Cyprus; 1999.
10. Hadjikakou K, Petridou L, Stylianou C. The academic and social inclusion of oral deaf and hard‐of‐hearing children in Cyprus secondary general education: investigating the perspectives of the stakeholders. Eur J Spec Needs Educ. 2008 Feb [cited 2019 Aug 29];23(1):17–29. Available from: http://www.tandfonline.com/doi/abs/10.1080/08856250701791211
11. Liasidou A. Inclusive education policies and the feasibility of educational change: the case of Cyprus. Int Stud Sociol Educ. 2007 Dec [cited 2019 Aug 23];17(4):329–47. Available from: http://www.tandfonline.com/doi/abs/10.1080/09620210701666972
12. Cypriot Ministry of Education and Culture. Code of Practice for Referring Children with Special Needs to the Special Education and Training District Committees. 2003.
13. Cypriot Ministry of Education and Culture. Primary Education Circulars. 2019.
14. Cypriot Ministry of Education and Culture. Primary Education Circulars. 2018. Available from: http://www.pi.ac.cy/pi/files/anakoinoseis/2018_2019/20190619_proairetika_diagnosi_anagkon.pdf
15. Cypriot Ministry of Education and Culture. Primary Education Circulars. 2016. Available from: http://www.pi.ac.cy/pi/files/anakoinoseis/2015_2016/20160610_diimero_ekp.pdf
16. Symeonidou S. Initial teacher education for inclusion: a review of the literature. Disabil Soc. 2017 Mar 16 [cited 2019 Aug 20];32(3):401–22. Available from: https://www.tandfonline.com/doi/full/10.1080/09687599.2017.1298992
17. Symeonidou S, Mavrou K. Problematising disabling discourses on the assessment and placement of learners with disabilities: can interdependence inform an alternative narrative for inclusion? Eur J Spec Needs Educ. 2019 Apr 25 [cited 2019 Aug 20];1–15. Available from: https://www.tandfonline.com/doi/full/10.1080/08856257.2019.1607661

**Luxembourg**

1. Luxembourgian Ministry of National Education. Act of 5 August 1963 on the reform of pre-school education and primary education. 1963. Available from: http://legilux.public.lu/eli/etat/leg/loi/1963/08/05/n4/jo
2. Luxembourgian Ministry of National Education. Law of 14 March 1973 establishing institutes and services of differentiated education. 1973. Available from: http://legilux.public.lu/eli/etat/leg/loi/1973/03/14/n1/jo
3. Luxembourgian Ministry of National Education. Grand-Ducal Decree of 4 March 1988 establishing an Institute for Autistic and Psychotic Children. 1988. Available from: http://legilux.public.lu/eli/etat/leg/agd/1988/03/04/n1/jo
4. Luxembourgian Ministry of National Education. Law of 10 January 1989 on 1. the resumption of differentiated education centers and services in certain municipalities, 2. amendment of the law of 14 March 1973 establishing institutes and educational services differentiated, 3. amendment of the law of 16. 1989. Available from: http://www.men.public.lu/fr/legislation/education-nationale/eleves-specifiques/ch6-loi-10-janvier-89.pdf
5. Luxembourgian Ministry of National Education. Law of 28 June 1994 amending and supplementing (a) the amended law of 10 August 1912 concerning the organization of primary education; (b) the amended Act of 14 March 1973 establishing differentiated education institutes and services; for the participatio. 1994. Available from: http://legilux.public.lu/eli/etat/leg/loi/1994/06/28/n1/jo
6. Luxembourgian Ministry of National Education. Law of 6 February 2009 on compulsory education. 2009 [cited 2019 Feb 14]. Available from: http://legilux.public.lu/eli/etat/leg/trep/2009/09/03/n1/jo
7. Luxembourgian Ministry of National Education. Law of 6 February 2009 on the organization of basic education. 2009 [cited 2019 Feb 14]. Available from: http://legilux.public.lu/eli/etat/leg/trep/2014/09/04/n2/jo
8. Luxembourgian Ministry of National Education. Law of 20 July 2018 establishing Centers of Competence in Specialized Psycho-Pedagogy for Inclusive Education. 2018. Available from: http://legilux.public.lu/eli/etat/leg/loi/2018/07/20/a664/jo
9. Luxembourgian Ministry of National Education. Grand-Ducal Regulation of 7 September 2018 on the Center for the Development of Children and Young People with Autism Spectrum Disorder. 2018. Available from: http://legilux.public.lu/eli/etat/leg/rgd/2018/09/07/a806/jo

**Slovenia**

1. National Assembly of the Republic of Slovenia. Pre-School Institutions Act. 1996. Available from: http://www.pisrs.si/Pis.web/pregledPredpisa?id=ZAKO447
2. National Assembly of the Republic of Slovenia. Primary School Act. 1996. Available from: http://www.pisrs.si/Pis.web/pregledPredpisa?id=ZAKO448
3. National Assembly of the Republic of Slovenia. Gymnasium Act. 1996. Available from: http://www.pisrs.si/Pis.web/pregledPredpisa?id=ZAKO450
4. Slovenian Ministry of Education and Sport. Decree on norms and standards for the education and training of children and youth with special needs. 1997. Available from: http://www.pisrs.si/Pis.web/pregledPredpisa?id=ODRE1448
5. Slovenian Ministry of Education and Sport. Special Education Program. 2014. Available from: http://www.mizs.gov.si/delovna_podrocja/direktorat_za_predsolsko_vzgojo_in_osnovno_solstvo/izobrazevanje_otrok_s_posebnimi_potrebami/programi/posebni_program_vzgoje_in_izobrazevanja/
6. Slovenian Ministry of Education and Sport. Decree amending and supplementing the Decree on norms and standards for the education and training of children and youth with special needs. 2001. Available from: http://www.pisrs.si/Pis.web/pregledPredpisa?id=ODRE1656
7. National Assembly of the Republic of Slovenia. Organization and Financing of Education Act (official consolidated text). 2001. Available from: http://www.pisrs.si/Pis.web/pregledPredpisa?id=ZAKO3592
8. National Assembly of the Republic of Slovenia. Disabled Persons Organizations Act. 2002. Available from: http://www.pisrs.si/Pis.web/pregledPredpisa?id=ZAKO1460
9. Slovenian Ministry of Education and Sport. Rules on norms and standards for the implementation of educational programs for children with special needs in elementary schools with an adapted program and institutions for the education and training of children with special needs. 2005. Available from: https://www.uradni-list.si/glasilo-uradni-list-rs/vsebina/2005-01-3293?sop=2005-01-3293
10. National Assembly of the Republic of Slovenia. Equalization of Opportunities for Persons with Disabilities Act. 2010. Available from: http://www.pisrs.si/Pis.web/pregledPredpisa?id=ZAKO4342
11. National Assembly of the Republic of Slovenia. Guidance of Children with Special Needs Act. 2011. Available from: http://www.pisrs.si/Pis.web/pregledPredpisa?id=ZAKO5896
12. Slovenian Ministry of Education,Science and Sport,. Rules on the organization and work methods of commissions for the placement of children with special needs. 2013. Available from: http://www.pisrs.si/Pis.web/pregledPredpisa?id=PRAV11834
13. Slovenian Ministry of Labor, Family and Social Affairs and Equal Opportunities,. Rules on criteria for claiming rights for children in need of special care. 2014. Available from: http://www.pisrs.si/Pis.web/pregledPredpisa?id=PRAV12241
14. Slovenian Ministry of Education, Science and Sport,. Decree on the adoption of the adapted primary school programme with equivalent education standard for children with autistic disorders. 2014. Available from: http://www.pisrs.si/Pis.web/pregledPredpisa?id=ODRE2283
15. Slovenian Ministry of Labor, Family, Social Affairs and Equal Opportunities,. Foundation for children and adults with autism - ZORA. 2016. Available from: http://www.pisrs.si/Pis.web/pregledPredpisa?id=ODLB1397
16. Slovenian Ministry of Labor, Family, Social Affairs and Equal Opportunities,. Protection Against Discrimination Act. 2016. Available from: http://www.pisrs.si/Pis.web/pregledPredpisa?id=ZAKO7273
17. Slovenian Ministry of Health. Resolution on the national mental health programme 2018-2028. 2018.
